# Outcomes and complications of the reamer irrigator aspirator versus traditional iliac crest bone graft harvesting: a systematic review and meta-analysis

**DOI:** 10.1186/s10195-021-00612-9

**Published:** 2021-12-01

**Authors:** Francesco Oliva, Filippo Migliorini, Francesco Cuozzo, Ernesto Torsiello, Frank Hildebrand, Nicola Maffulli

**Affiliations:** 1 Department of Musculoskeletal Disorders, Faculty of Medicine and Surgery, University of Salerno, 84084 Baronissi, Italy; 2 Clinica Ortopedica, Ospedale San Giovanni di Dio e Ruggi d’Aragona, 84131 Salerno, Italy; 3 Department of Orthopaedic and Trauma Surgery, RWTH University Hospital Aachen, Pauwelsstraße 31, 52074 Aachen, Germany; 4 Centre for Sports and Exercise Medicine, Barts and The London School of Medicine and Dentistry, Mile End Hospital, 275 Bancroft Road, London, E1 4DG England; 5 School of Pharmacy and Biotechnology, Keele University School of Medicine, Thornburrow Drive, Stoke-on-Trent, England

**Keywords:** Reamer, Irrigator, Aspirator, RIA, Iliac, Crest, Bone, Graft, ICBG, Nonunion, Autologous, Bone, Graft

## Abstract

**Background:**

The reamer irrigator aspirator (RIA) is a relatively recent device that is placed in the medullary canal of long bones to harvest a large volume of bone marrow, which is collected in a filtered canister. This study compares outcomes and complications of the RIA versus a traditional iliac crest bone graft (ICBG) for the treatment of bone defects.

**Methods:**

This meta-analysis was conducted according to the PRISMA guidelines. The Embase, Google Scholar, PubMed, and Scopus databases were accessed in June 2021. All clinical trials comparing the RIA and ICBG with a minimum of 6 months follow-up were included.

**Results:**

Data from 4819 patients were collected. The RIA group demonstrated lower site pain (*P* < 0.0001), fewer infections (*P* = 0.001), and a lower rate of adverse events (*P* < 0.0001). The ICBG group demonstrated a greater rate of bone union (*P* < 0.0001). There was no difference between groups in VAS (*P* = 0.09) and mean time to union (*P* = 0.06).

**Conclusion:**

The current evidence supports the use of the RIA, given its low morbidity and short learning curve.

## Introduction

Autologous bone grafting is a commonly performed procedure [1]. Arthrodesis, long bone nonunion, osteomyelitis, and regenerative strategies for osteochondral defects are some of the surgical procedures in which autologous bone grafting is indicated [2–7]. An iliac crest bone graft (ICBG) is commonly used to obtain autologous bone for grafting [8, 9]. Usually, a skin incision is made parallel to the iliac crest and the iliac spine is exposed subperiosteally, with the periosteum and muscle fascia on the medial edge of the crest preserved [10, 11]. This harvest typically involves a horizontal cut through the outer cortex of the iliac crest followed by the crest reflection medially, without disturbing the attachment site of the abdominal muscles [12]. After sufficient graft material has been harvested, the iliac crest is sutured [13]. Autologous crest bone grafting is not without complications, the most common being pain at the harvest site, wound infections, fractures, and hematomas [9, 14–16]. A relatively recent harvesting technique includes the use of the reamer irrigator aspirator (RIA) [17]. This new device has the advantage of allowing large amounts of autologous bone graft to be harvested from the medullary canal of a long bone with a lower rate of morbidities and complications [18, 19]. After introducing the RIA and performing combined reaming and aspiration, the graft is collected inside a filtered canister [20]. This technique is versatile and has a short learning curve, suggesting that it represents a valid alternative to traditional techniques [17].

This study compares the ICBG and RIA, seeking to demonstrate the noninferiority of RIA as a harvesting technique. The primary outcomes were the visual analogue scale (VAS) score and time to union. The secondary outcome includes the most common complications, such as donor site pain, fracture, infections, and hematoma/seroma.

## Materials and methods

### Search strategy

This systematic review was conducted according to the Preferred Reporting Items for Systematic Reviews and Meta-Analyses: the PRISMA guidelines [21]. The PICOT framework was followed:

- P (problem): long bones non-union, arthrodesis, osteomyelitis, maxillofacial surgery;
- I (intervention): autologous bone grafting;
- C (comparison): RIA vs ICBG;
- O (outcomes): PROMs, time to union, rate of union, complications;
- T (timing): ≥ 6 months follow-up.

### Data source and extraction

Two authors (FC; ET) independently performed the literature search in June 2021. PubMed and Google Scholar were accessed. Embase and Scopus were successively accessed to identify further articles. The following keywords were used in combination: “autologous,” “iliac,” “crest,” “bone,” “marrow,” “graft,” “reamer,” “irrigator,” “aspirator,” “posterior,” “anterior,” “ACBG,” “PCBG,” “ICBG,” “RIA,” “device,” “technique,” “long,” “bone,” “non-union,” “arthrodesis,” “osteomyelitis,” “PROMs,” “complications,” “morbidity,” “donor,” “site,” “surgery,” “harvesting,” “collection,” “medullary,” “canal,” and “invasiveness.” If the title and abstract matched the topic, the full-text article was accessed. The bibliographies of the full-text articles were screened for inclusion. Disagreements were resolved by a third author (**).

### Eligibility criteria

All clinical studies comparing autologous crest bone grafting using the anterior or posterior harvesting technique with the RIA technique were accessed. Given the authors’ language capabilities, articles in English, German, Italian, French, and Spanish were eligible. Level I–IV evidence (according to the Oxford Centre of Evidence-Based Medicine) was considered. Only studies published in peer-reviewed journals were considered. Editorials, systematic reviews, meta-analyses, technical notes, narrative reviews, expert opinions, and letters were excluded. Animal, biomechanical, and cadaveric studies were also excluded. Only articles reporting a minimum of 6 months follow-up were included. Studies involving skeletally immature patients were not eligible. Only articles reporting quantitative data under the outcomes of interest were considered for inclusion.

### Outcomes of interest

Two authors (**; **) independently performed data extraction. The following data were collected: generalities (author, year, type of study), demographic baseline (number of samples, mean age), mean follow-up, mean BMI, indication for surgical intervention (long bone nonunion, spinal surgery, osteomyelitis), and harvesting site. The following outcomes of interest were collected: visual analogue scale (VAS) and time to union (mean).

### Methodological quality assessment

Methodological quality assessment was performed by a single author (**) through the Coleman Methodology Score (CMS). The CMS is a reliable and validated tool to evaluate the methodological quality of systematic reviews and meta-analyses [22]. This score analyses the included articles, evaluating the population size, length of follow-up, surgical approach, study design, description of diagnosis, surgical technique, and rehabilitation. Outcome criteria assessment and the subject selection process were also evaluated. The quality of each study was scored between 0 (poor) and 100 (excellent), with a value of > 60 considered satisfactory.

### Statistical analysis

The statistical analysis was performed by a single author (**) using the IBM SPSS software, version 25. Baseline comparability was assessed through the mean difference (MD) and the unpaired* t*-test, with values of *P* > 0.1 considered satisfactory. For the noncomparative studies included in the systematic review, the MD was used for continuous variables and the odds ratio (OR) for dichotomic data. The* t*-test and the* χ*^2^ test were performed, respectively, with values of *P* < 0.05 considered statistically significant. The confidence interval (CI) was set at 95%. Comparative studies were included in the meta-analyses. The meta-analyses were performed using Review Manager 5.3 software (The Nordic Cochrane Collaboration, Copenhagen). For continuous data, the inverse variance method was used, with MD as the effect measure. For dichotomic data, the Mantel–Haenszel method was used, with OR as the effect measure. A fixed model analysis was used as default in all the comparisons. Heterogeneity was evaluated through the Higgins *I*^2^ test. *I*^2^ was interpreted according to the* Cochrane Handbook for Systematic Reviews of Interventions* (http://www.cochrane-handbook.org) as follows: 0–40%, poor heterogeneity; 30–60%, moderate heterogeneity; 50–90%, substantial heterogeneity; 75–100%, considerable heterogeneity. If *I*^2^ > 60%, we switched to a random model analysis. Values of *P* < 0.05 were considered statistically significant.

## Results

### Search results

**Fig. 1:**
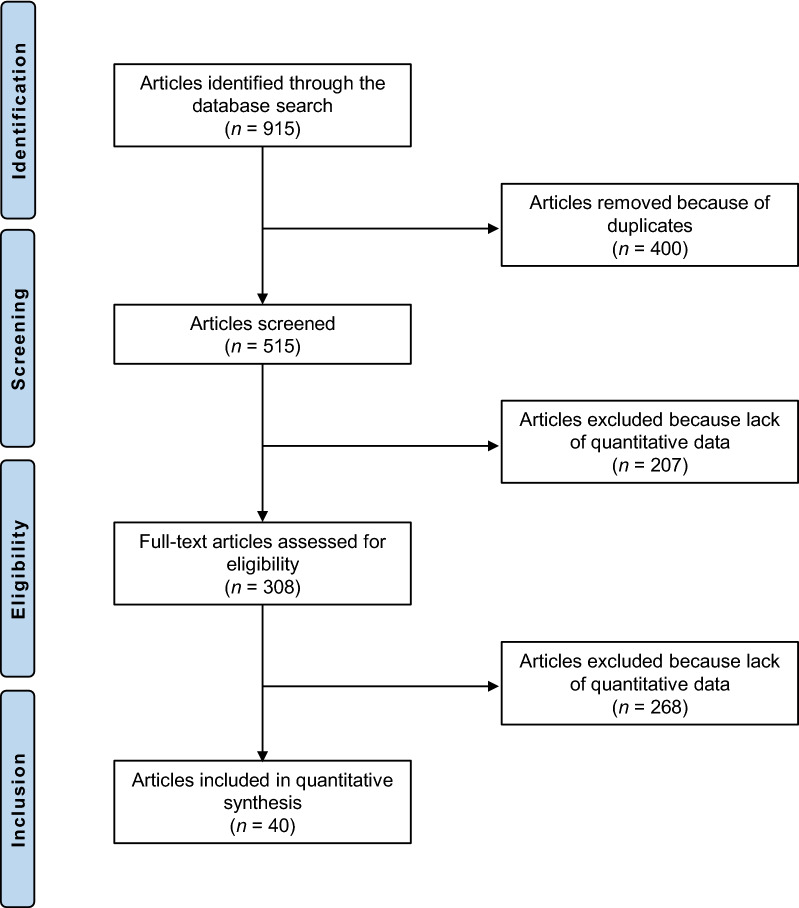
Flow chart of the literature search

The literature search resulted in 915 articles. After the removal of duplicates (*N* = 400), a further 475 articles were found to be ineligible for the following reasons: study design (*N* = 365); language limitation (*N* = 17); short follow-up (*N* = 38); lacking quantitative data under the endpoints of interest (*N* = 49); cadaveric studies (*N* = 6). Finally, 40 comparative studies were included: one randomized controlled trial (RCT) and 10 prospective and 29 retrospective clinical studies. The literature search results are shown in Fig. 1.

### Methodological quality assessment

The CMS identified limitations and strengths of the present study. The study size and length of the follow-up were adequate. The surgical approach and diagnosis were well described in most articles.

Outcome measures and timing of assessment were frequently defined, providing moderate reliability. The procedures used for assessing outcomes and selecting subjects were often biased and poorly described. The CMS was 67 points, indicating that the methodological quality was fair. The CMS results are shown in Table 1.

**Table 1:** Methodological quality assessment

Authors, year	Part A: Only one score to be given for each of the seven sections							Part B: Scores may be given for each option in each of the three sections if applicable										
	Study size	Mean follow-up	Surgical approach	Type of study	Description of diagnosis	Description of surgical technique	Description of postoperative rehabilitation	Outcome criteria				Procedure used to assess outcomes			Description of subject selection process			
								Outcome measures clearly defined	Timing of outcome assessment clearly stated	Use of outcome criteria that have reported reliability	General health measure included	Participants recruited	Investigator independent of surgeon	Written assessment	Completion of assessment by patients themselves with minimal investigator assistance	Selection criteria are reported and unbiased	Reported recruitment rate > 80%	Reported recruitment rate < 80%
Alhmann et al., 2002 [9]	7	7	7	10	5	5	0	2	2	3	3	5	0	0	3	5	5	0
Almaiman et al., 2013 [16]	10	4	10	0	5	10	0	2	2	3	3	5	0	0	3	5	5	0
Banwart et al., 1995 [39]	10	10	10	0	5	5	0	2	2	3	3	5	0	0	3	5	5	0
Belthur et al., 2008 [17]	7	4	7	0	5	10	0	2	2	3	3	5	0	3	3	5	5	0
Beirne et al., 1996 [31]	10	4	10	0	5	10	0	2	2	3	3	5	0	0	3	5	5	0
Burstein et al., 2000 [40]	7	4	10	0	5	10	0	2	2	3	3	5	0	0	3	5	5	0
Calori et al., 2014 [2]	4	0	7	0	5	5	5	2	2	3	3	5	0	0	3	5	5	0
Carlock et al., 2019 [35]	10	4	10	0	5	5	0	2	2	3	3	5	0	3	3	5	5	0
Conway et al., 2014 [41]	0	0	10	0	5	10	0	2	2	3	3	5	0	0	3	5	5	0
David et al., 2003 [42]	10	4	10	0	5	10	0	2	2	3	3	5	0	3	3	5	5	0
Dawson et al., 2014 [24]	10	7	7	15	5	10	0	2	2	3	3	5	0	0	3	5	5	0
Delawi et al., 2007 [43]	7	10	10	0	5	10	0	2	2	3	3	5	0	0	3	5	5	0
Deorio et al., 2005 [27]	10	10	10	0	5	10	5	2	2	3	3	5	0	0	3	5	5	0
Dimar et al., 2009 [32]	10	4	10	0	5	5	0	2	2	3	3	5	0	0	3	5	5	0
Fernyhough et al., 1992 [44]	10	4	10	0	5	10	0	2	2	3	3	5	0	0	3	5	5	0
Finkemeir et al., 2010 [45]	4	4	10	0	5	10	0	2	2	3	3	5	0	0	3	5	5	0
Goulet et al., 1997 [29]	10	7	10	0	5	5	0	2	2	3	3	5	0	3	3	5	5	0
Han et al., 2015 [18]	7	7	10	0	5	10	0	2	2	3	3	5	0	0	3	5	5	0
Haubruck et al., 2018 [46]	10	10	10	0	5	10	0	2	2	3	3	5	0	3	3	5	5	0
Hill et al., 1999 [47]	7	7	10	0	5	5	0	2	2	3	3	5	0	0	3	5	5	0
Kanakaris et al., 2011 [37]	4	0	10	10	5	5	0	2	2	3	3	5	0	0	3	5	5	0
Kim et al., 2009 [48]	10	4	10	10	5	5	0	2	2	3	3	5	0	0	3	5	5	0
Kusnezov et al., 2017 [49]	4	4	10	0	5	10	0	2	2	3	3	5	0	0	3	5	5	0
Le Baron et al., 2019 [23]	7	7	7	10	5	10	0	2	2	3	3	5	0	0	3	5	5	0
Loeffler et al., 2012 [50]	7	4	10	10	5	10	0	2	2	3	3	5	0	0	3	5	5	0
Mccall et al., 2010 [20]	4	7	10	0	5	10	0	2	2	3	3	5	0	0	3	5	5	0
Marchand et al., 2017 [26]	10	4	7	0	5	10	0	2	2	3	3	5	0	0	3	5	5	0
Merrit et al., 2010 [51]	7	4	10	0	5	10	0	2	2	3	3	5	0	0	3	5	5	0
Metsemakers et al., 2019 [52]	7	10	10	0	5	10	0	2	2	3	3	5	4	0	3	5	5	0
Mirovski et al., 2000 [53]	7	4	7	10	5	10	0	2	2	3	3	5	0	3	3	5	5	0
Nodzo et al., 2014 [25]	4	0	7	0	5	5	0	2	2	3	3	5	0	0	3	5	5	0
On Salawu et al., 2017 [36]	7	4	10	10	5	10	0	2	2	3	3	5	0	3	3	5	5	0
Pollock et al., 2008 [28]	7	4	10	0	5	10	0	2	2	3	3	5	0	0	3	5	5	0
Qvick et al., 2013 [3]	10	7	10	0	5	10	0	2	2	3	3	5	0	0	3	5	5	0
Robertson et al., 2001 [54]	10	4	10	10	5	10	0	2	2	3	3	5	4	0	3	5	5	0
Schizas et al., 2009 [55]	7	4	10	10	5	5	0	2	2	3	3	5	0	0	3	5	5	0
Schwartz et al., 2009 [56]	10	7	10	10	5	5	0	2	2	3	3	5	0	3	3	5	5	0
Silber et al., 2003 [33]	10	7	10	0	5	10	0	2	2	3	3	5	0	0	3	5	5	0
Westrich et al., 2001 [30]	10	0	10	0	5	10	0	2	2	3	3	5	0	0	3	5	5	0
Younger et al., 1989 [8]	10	0	10	0	5	5	0	2	2	3	3	5	0	0	3	5	5	0

#### Risk of publication bias

**Fig. 2:**
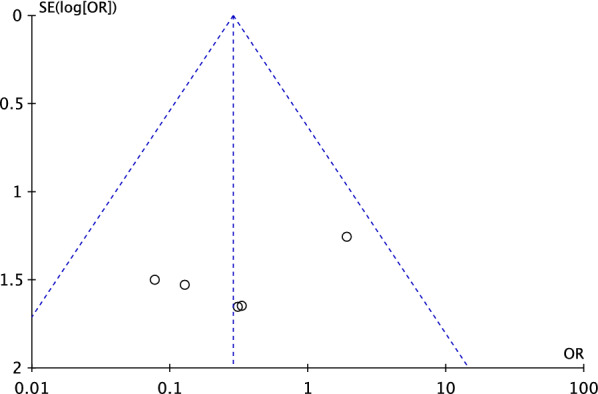
Funnel plot

A funnel plot of the most commonly reported outcome (infections) was used to evaluate the risk of publication bias. The plot evidenced good symmetry, and all the referrals were located within the pyramid. Thus, the funnel plot indicated a low risk of publication bias (Fig. 2).

### Patient demographics

Data from 4819 patients were collected, 1908 of whom were women. There was comparability between the two groups in terms of mean age, mean BMI, and mean harvest volume. Study generalities and patient demographics are shown in detail in Table 2, while the results of the baseline comparison are reported in Table 3.

**Table 2:** Generalities and patient baselines of the included studies

Authors, year	Journal	Type of study	CMS	Follow-up (months)	Treatment	Patients (*n*)	Mean age (years)	Female (*n*)
Alhmann et al., 2002 [9]	*J Bone Joint Surg Am*	Retrospective	67	60.0	ICBG	88	46.2	13
					ICBG			
Almaiman et al., 2013 [16]	*Craniomaxillofac Trauma Reconstr*	Retrospective	67	12.0	ICBG	372		172
Banwart et al., 1995 [39]	*Spine*	Retrospective	68	66.0	ICBG	180	26.3	115
Belthur et al., 2008 [17]	*Clin Orthop Relat Res*	Retrospective	64	9.1	RIA	41	44.9	18
				20.2	ICBG	40	46.4	17
Beirne et al., 1996 [31]	*Int J Oral Maxilofac Surg*	Retrospective	67	12.0	ICBG	137		60
Burstein et al., 2000 [40]	*Plastic Reconstr Surg*	Retrospective	60		ICBG	55	11.2	22
Calori et al., 2014 [2]	*Injury*	Retrospective	54	12.0	RIA	35	50.17	12
					ICBG	35	53.62	14
Carlock et al., 2019 [35]	*J Am Acad Orthop Surg*	Retrospective	65	12.0	ICBG	242	58.3	107
Conway et al., 2014 [41]	*Orthopedics*	Retrospective	51	6.5	RIA	7	45.0	3
David et al., 2003 [42]	*J Spinal Disord Tech*	Retrospective	70	26.5	ICBG	107	42.75	43
Dawson et al., 2014 [24]	*J Orthop Trauma*	RCT	82	56.9	RIA	56	41.3	23
					ICBG	57	47.4	22
Delawi et al., 2007 [43]	*Spine*	Retrospective	70	87.6	ICBG	71	47.6	22
Deorio et al., 2005 [27]	*Foot Ankle Int*	Retrospective	78	74.4	ICBG	134		
Dimar et al., 2009 [32]	*Spine* J	Retrospective	62	24.0	ICBG	194	52.3	
Fernyhough et al., 1992 [44]	*Spine*	Retrospective	67	12.0	ICBG	147		50
Finkemeir et al., 2010 [45]	*Orthop Clinic North Am*	Retrospective	61	18.0	RIA	23	50.0	10
Goulet et al., 1997 [29]	*Clinic Orthop Relat Res*	Retrospective	61		ICBG	170	41.0	60
Han et al., 2015 [18]	*Injury*	Retrospective	67	54.0	RIA	57		3
Haubruck et al., 2018 [46]	*PloS One*	Retrospective	75	75.0	RIA	306	54.0	113
Hill et al., 1999 [47]	*Aust N Z J Surg*	Retrospective	62	37.0	ICBG	73	38.0	31
Kanakaris et al., 2011 [37]	*Injury*	Prospective	62	6.0	RIA	42	45.5	14
Kim et al., 2009 [48]	*Spine* J	Prospective	72	12.0	ICBG	110	50.4	56
Kusnezov et al., 2017 [49]	*SICOT J*	Retrospective	61	13.3	RIA	15	41.1	5
Le Baron et al., 2019 [23]	*Orthop Traumatol Surg Res*	Prospective	74	22.1	RIA	30	38.9	9
				56.7	ICBG	29	35.3	6
Loeffler et al., 2012 [50]	*J Bone Joint Surg Am*	Prospective	74	12.0	ICBG	92		31
Mccall et al., 2010 [20]	*Orthop Clinic North Am*	Prospective	64	48.0	RIA	21	30.6	8
Marchand et al., 2017 [26]	*J Orthop Trauma*	Retrospective	62	13.0	RIA	61	51.0	50
					ICBG	47	54.0	
Merrit et al., 2010 [51]	*Spine*	Retrospective	64	24.0	ICBG	92	62.0	60
Metsemakers et al., 2019 [52]	*Eur J Trauma Emerg Surg*	Retrospective	72	84.0	RIA	72	45.4	23
Mirovski et al., 2000 [53]	*Spine*	Prospective	72	24.0	ICBG	60	39.8	
Nodzo et al., 2014 [25]	*Int Orthop*	Retrospective	47		RIA	29	49.4	21
					ICBG	27	49.3	21
On Salawu et al., 2017 [36]	*Malays Orthop J*	Prospective	77	13.0	ICBG	86	40.8	33
Pollock et al., 2008 [28]	*Eur Spine j*	Retrospective	64	19.8	ICBG	77	46.1	47
Qvick et al., 2013 [3]	*Injury*	Retrospective	70	48.0	RIA	204	50.0	88
Robertson et al., 2001 [54]	*Spine*	Prospective	78	12.0	ICBG	106	47.4	72
Schizas et al., 2009 [55]	*Int Orthop*	Prospective	69	36.0	ICBG	59		
Schwartz et al., 2009 [56]	*Health Qual Life Outcomes*	Prospective	78	42.9	ICBG	170	51.1	
Silber et al., 2003 [33]	*Spine*	Retrospective	70	48.0	ICBG	134		75
Westrich et al., 2001 [30]	*J Orthop Trauma*	Retrospective	63		RIA	390	47.9	183
Younger et al., 1989 [8]	*J Orthop Trauma*	Retrospective	58	11.0	ICBG	239	33.0	86

*RIA* reamer irrigator aspirator, *ICBG* iliac crest bone graft, *RCT* randomized controlled trial

**Table 3:** Patient baseline demographics of the included studies

Endpoint	ICBG (*N* = 3430)	RIA (*N* = 1389)	*P*
Mean age	44.4 ± 10.9	45.7 ± 5.9	0.6
Mean BMI	27.9 ± 2.6	28.6 ± 3.3	0.8
Harvest volume (mean)	38.7 ± 15.7	47.6 ± 12.1	0.2

### Outcomes of interest

There was no difference between the groups in terms of VAS (*P* = 0.09) and mean time to union (*P* = 0.06) (Table 4).

**Table 4:** Comparison of VAS and mean time to union

Endpoint	ICBG (*N* = 3430)	RIA (*N* = 1389)	MD	*P*
VAS	2.3 ± 1.5	1.7 ± 2.4	0.6	0.09
Time to union (mean)	11.5 ± 1.9	7.2 ± 1.6	4.3	0.06

### Complications

The RIA group demonstrated lower site pain (OR 13.2; 95% CI 8.4926–20.4135; *P* < 0.0001), a lower incidence of infection (OR 2.85; 95% CI 1.5060–5.4168; *P* = 0.001), and a lower rate of adverse events (OR 5.80; 95% CI 3.2118–10.50; *P* < 0.0001). The ICBG group demonstrated a greater rate of bone union (OR 17.28; 95% CI 12.8770–23.1941; *P* < 0.0001) compared to RIA. No difference was found in the fracture rate (*P* = 0.7) and the hematoma/seroma rate (*P* = 0.4). These results are shown in detail in Table 5.

**Table 5:** Complications

Endpoint	ICBG	RIA	OR	95% CI	*P*
Site pain	38.7% (674 of 1742)	4.6% (22 of 481)	13.16	8.4926–20.4135	< 0.0001
Fracture	1.0% (4 of 407)	1.2% (7 of 567)	0.79	0.2309–2.7306	0.7
Infection	5.0% (75 of 1493)	1.8% (11 of 605)	2.85	1.5060–5.4168	0.001
Hematoma/seroma	1.4% (17 of 1204)	0.9% (4 of 462)	1.63	0.5489–4.8995	0.4
Union	88.3% (477 of 540)	81.6% (315 of 386)	17.28	12.8770–23.1941	< 0.0001
Adverse event	22.3% (327 of 1467)	4.7% (12 of 255)	5.80	3.2118–10.5048	< 0.0001

### Meta-analyses

**Fig. 3:**
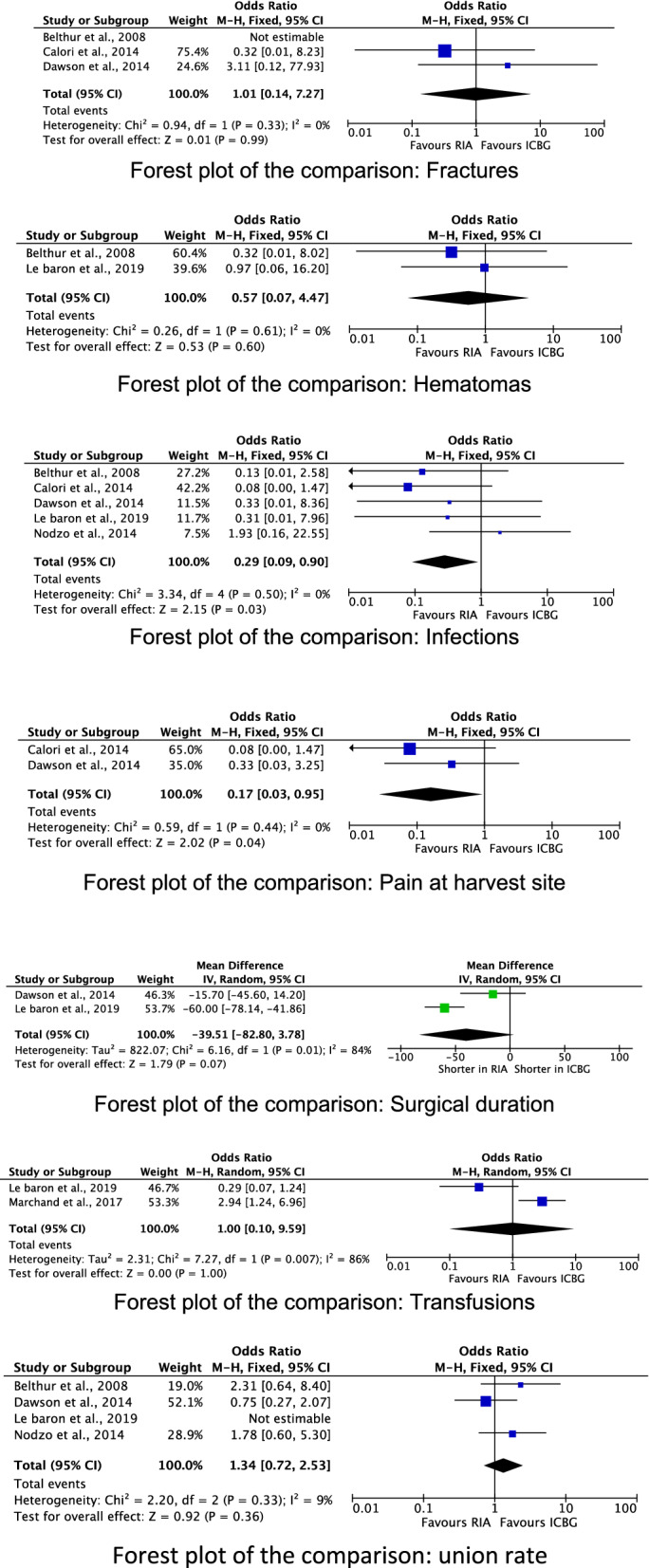
Meta-analyses

Six studies that directly compared the RIA to ICBG were included in the meta-analysis [2, 17, 23–26]. A total of 487 patients were included, 213 of whom were female. The mean follow-up was 12.5 ± 0.7 months. The mean age was 46.8 ± 5.8, and the mean BMI was 27.6 ± 3.1 kg/m^2^. Comparability was found at baseline in terms of age and BMI (*P* > 0.1). Similarity was found in the length of the surgical intervention (*P* = 0.07), the transfusion rate (*P* = 1.0), the fracture rate (*P* = 1.0), the hematoma rate (*P* = 0.6), and the union rate (*P* = 0.4). The RIA demonstrated lower painful harvest site (OR 0.17; 95%CI 0.03–0.95; *P* = 0.04) and infection (OR 0.29; 95%CI 0.09–0.90; *P* = 0.03) rates. These results are shown in greater detail in Fig. 3.

## Discussion

According to the main findings of the present study, the RIA was associated with less morbidity than the ICBG. There was no difference in terms of VAS and mean time to union between the two groups. The RIA demonstrated a lower incidence of harvest site pain, with only 22 of 481 patients reporting this symptom, in contrast to the ICBG cohort, for which there were 674 cases of site pain in 1742 patients. Belthur et al. [17] investigated the intensity and frequency of donor site pain. In the first 48 postoperative hours, the total pain score was higher for the ICBG than for the RIA cohort; in the period between 48 h and three months postoperatively, site pain was lower in the RIA group; after three months, the RIA group did not report donor site pain, unlike the ICBG group [17]. Donor site pain is one of the most common complications in all bone marrow harvesting procedures [16, 27–30]. Moreover, the ICBG procedure may impair sexual function, limit daily activities, and expose patients to infections [31–34]. Calori et al. reported no site infections in the RIA cohort (0/35) but a site infection rate of 14% (5/35) in the ICBG group [2]. Similarly, Belthur et al. noted that there were no infections in the RIA group (0/41) but that 8% (3/40) of the donor sites were infected in the ICBG cohort [17].

No difference was found between the groups in fracture or hematoma/seroma occurrence. The groups were similar in terms of surgery duration and transfusion rate, although the RIA is often reported in the literature to produce greater blood loss [2, 18, 26]. Overall, the adverse event rate was lower in the RIA cohort. Regarding the union rate, our results are controversial. Although the overall union rate was statistically significantly greater in the ICBG cohort (88.3% versus 81.6%), the meta-analysis of comparative studies demonstrated no significant difference between the cohorts. In this context, our findings are not fully generalizable, and no reliable conclusions can be inferred. The current evidence is controversial. Dawson et al. reported a higher union rate in the ICBG cohort compared to the RIA group [24]. Carlock et al. reported a high union rate after ICBG, with 232 unions in 242 treated patients [35]. Furthermore, On Salawu et al. reported a higher union rate following ICBG, with 81 unions in 86 patients [36]. In this regard, the data in the literature are controversial, because the RIA group is characterized by a higher union rate [17, 25]. Han et al. reported 50 unions in 57 patients after the RIA procedure [18]. Kanakaris and colleagues reported 41 unions in 42 procedures after RIA [37]. Conversely, Le Baron et al. reported nearly the same union rates in these two groups [23]. Dimitriou et al. compared the main complications after RIA use or after autologous crest bone grafting, and described two different access sites on the iliac crest: anterior and posterior [14]. The use of the RIA as a harvesting method seems to be characterized by lower rates of infection, hematoma formation, and fracture [14]. Cox et al. reported that the RIA appears relatively safe, with a lower morbidity rate than ICBG. Moreover, when complications occur, patients treated with the RIA respond better than those treated with an ICBG [38].

Our study is not free of limitations. The retrospective design of most of the included studies is an important limitation. Unfortunately, only one study was a randomized clinical trial [24], which represents an important source of selection bias. The postoperative rehabilitation was seldom described, and the follow-up was limited in most of the studies. The description of the surgical technique used was fair in several studies, representing a further limitation. Given the limited data available, and to increase the data pooling, anterior and posterior iliac crest bone grafting were not analyzed separately. However, previous evidence demonstrated that posterior and anterior ICBG produce similar outcomes [8, 9]. Finally, it is strongly recommended that further high-quality clinical trials that provide long-term follow-up should be performed to establish whether RIA can be considered the new gold standard.

## Conclusion

Current evidence supports the use of the RIA, given its lower morbidity and shorter learning curve than ICBG. The RIA should become the new gold standard technique for bone marrow harvesting, but other clinical studies with long follow-ups are needed to prove it.

## Data Availability

The data underlying this article are available in the article and in its online supplementary material.

## Abbreviations

RIA: Reamer irrigator aspirator
ICBG: Iliac crest bone graft
BMI: Body mass index
VAS: Analogue scale
CMS: Coleman Methodology Score
MD: Mean difference
OR: Odds ratio
CI: Confidence interval (CI)
